# Can Beta-Lactam Allergy De-Labeling Strategies Safely Empower Geriatric Care?

**DOI:** 10.3390/jcm14103476

**Published:** 2025-05-15

**Authors:** Gal Goldhaber, Ronit Confino-Cohen, Idit Lachover-Roth, Anat Cohen-Engler, Saray Sity-Harel, Yossi Rosman

**Affiliations:** 1 School of Medicine, Tel-Aviv University, Tel-Aviv 69978, Israel; gal.goldhaber@clalit.org.il (G.G.); ronitco@clalit.org.il (R.C.-C.); idit.roth@clalit.org.il (I.L.-R.); anat.cohenengler@clalit.org.il (A.C.-E.); yossi.rosman@clalit.org.il (Y.R.); 2 Geriatric Department, Meir Medical Center, Kfar Saba 4428163, Israel; 3 Allergy and Clinical Immunology Unit, Meir Medical Center, Kfar Saba 4428163, Israel

**Keywords:** elders, beta-lactam, penicillin, drug allergy

## Abstract

**Background**: Drug allergies constitute a significant health concern among the elderly, with beta-lactam (BL) antibiotics among the most frequently implicated agents. Nevertheless, data regarding the safety and efficacy of BL allergy de-labeling in this population remain scarce. This study aimed to evaluate the safety and efficacy of BL allergy assessment in a cohort of geriatric patients carrying BL allergy labels. **Methods**: We conducted a retrospective study, including patients aged >65 years who were referred for BL allergy evaluation at the Allergy Unit of Meir Medical Center. Patients underwent comprehensive anamnesis, skin testing, and, when indicated, oral challenge. Those successfully de-labeled were followed longitudinally to assess subsequent BL use and clinical outcomes. **Results**: Between 2009 and 2019, 166 elderly patients with suspected BL allergies were evaluated. A BL allergy was ruled out in 145 patients (87.3%). Sixteen patients (9.6%) were diagnosed with immediate-type hypersensitivity, 2.4% of patients had severe delayed-type hypersensitivity reactions, and one patient (0.6%) had a benign rash. The evaluation process was safe, with no severe reactions occurring during oral challenges, and no patient required hospitalization or epinephrine administration. A long-term follow up was available for 106 patients; among them, 38 (35.8%) received subsequent treatment with the previously suspected BL agent, without any reports of immediate or severe delayed reactions. **Conclusions**: Beta-lactam allergy de-labeling is safe and effective in the elderly and supports the critical role of allergy evaluation in this population. Enhanced awareness and implementation of de-labeling protocols in geriatric patients are warranted.

## 1. Introduction

Drug hypersensitivity reactions (HSRs) are classified based on timing, mechanism, and clinical presentation. Immediate reactions, typically occurring within one to six hours of exposure, are often IGE mediated but may also result from direct mast cell activation via the MRGPRX2 receptor. Delayed reactions evolve over days to weeks and range from benign exanthems to severe cutaneous adverse reactions (SCARs) such as DRESS, AGEP, and SJS/TEN mediated by drug-specific T cells [1].

People over the age of 65 represent the fastest-growing demographic in the Western world population. By 2030, it is projected that they will comprise approximately 20% of the global population [2]. Drug allergies constitute a significant health concern in this population, owing to factors such as polypharmacy, multiple co-morbidities, fragility, and cognitive decline, which can impede accurate medical history-taking [3]. Beta-lactam (BL) antibiotics are among the most commonly reported causes of drug allergies, both in the general population and specifically among the elderly [3,4,5]. The true incidence of BL allergies remains unknown. It is estimated that up to 15% of the general population carries a documented BL allergy label. However, these labels often fail to differentiate true allergies from side effects or to classify allergic reactions by type (immediate, delayed, benign, or delayed severe). In many cases, the labeling was made erroneously, sometimes decades ago.

In addition, according to various studies, approximately 80% of patients with a penicillin allergy lose their sensitivity within 5–10 years of the initial allergic reaction [6,7].

The negative consequences of incorrect BL allergy labeling are well documented, affecting both the general and elderly populations. These consequences include the use of less effective second-line antibiotics, an increased risk of medication side effects, the emergence of drug-resistant bacteria, prolonged hospital stays, and a diminished quality of life due to concerns about potential allergic reactions [8,9,10]. While the safety and efficacy of BL allergy de-labeling are well established in general and pediatric populations, data regarding the geriatric population are scarce [1,11,12,13,14].

Our objective was to evaluate the safety and efficacy of BL allergy assessment in a substantial cohort of elderly patients carrying a BL allergy label.

## 2. Methods

### 2.1. Patients

This retrospective study included patients aged 65 years and older who were referred for BL allergy evaluation at the Allergy and Clinical Immunology Unit of the Meir Medical Center between 2009 and 2019. All patients underwent a comprehensive anamnesis, conducted by an allergy and clinical immunology specialist, which included predefined clinical questions regarding the suspected culprit drug, type of reaction, timing, and treatment required. Available medical records were reviewed for antibiotic usage between the allergic reaction and the current evaluation period. In rare cases, when the exact culprit drug was not listed in the patients’ medical file, it was presumed to be Penicillin VK, the sole penicillin available in Israel until the 1980s. Based on the collected information, patients were categorized as having nonallergic side effects, immediate reactions, delayed benign reactions (e.g., drug eruption), or severe delayed reactions (e.g., Stevens–Johnson syndrome, toxic epidermal necrolysis, drug-related eosinophilia with systemic symptoms, or acute generalized exanthematous pustulosis). Patients suspected of severe reactions were excluded from further evaluation and advised to permanently avoid BL antibiotics. Patients with a clinical history suggestive of side effects (e.g., abdominal pain, diarrhea, headache, etc.) were not subjected to skin tests (STs) or oral challenges. Their BL allergy labels were recommended for removal without further testing, and they were advised to use BL as needed.

Until 2015, all patients with suspected BL allergies (excluding those with suspected side effects) underwent skin tests, regardless of clinical suspicion. Following 2015, it was published by us and others that a direct oral challenge to BL is safe and efficient when the anamnesis is consistent with a late benign reaction [11,12,15]. Since then, only patients with suspected immediate reactions underwent prick and intradermal skin tests before an oral challenge. Patients with suspected late benign reactions underwent a direct oral challenge, with no need for skin testing. Skin tests included 0.04 mg/mL penicilloyl-poly-lysine (1:10 and 1:1), 0.5 mg/mL minor determinants mixture (1:10 and 1:1), 20 mg/mL amoxicillin (1:10 and 1:1) (all produced by Diater, Madrid, Spain), and 10,000 U/mL penicillin G (Teva, Israel). If the culprit BL was different, patients were also tested (prick and intradermally) with the relevant drug: 20 mg/mL amoxicillin–clavulanic acid (Augmentin by GSK, Brentford, UK), 2 mg/mL cefuroxime (Zinnat by GSK), 2.8 mg/mL ceftriaxone (Rocephine, Hoffman-La Roche, Basel, Switzerland), and 1 mg/mL cefazolin (Kefazin, Vitamed, Israel). Histamine phosphate (histatrol 2.75 mg/mL for intradermal ST and 0.275 mg/mL for prick ST, by ALK, Washington, NY) and phenol saline (ALK) served as positive and negative controls, respectively [16]. STs were considered positive when the largest diameter wheal was ≥3 mm compared to the negative control in the presence of a flare.

### 2.2. Oral Challenges

Until 2015, an oral challenge was recommended only following a negative ST. As mentioned above, since 2015, an oral challenge has been conducted directly when a delayed-type allergy was suspected, or after a negative skin test when an immediate allergy was suspected [11,12]. The oral challenge was administered using the suspected culprit BL. In cases where the initial culprit BL was unknown, the challenge was conducted with amoxicillin, which is the most common BL in use to date.

Patients received an initial dose equivalent to 1/10 of the single recommended dose. After one hour, provided there were no adverse reactions and following a thorough medical evaluation, patients were administered the full single dose and observed for an additional 2 h. Upon medical decision, in complex cases, a more graded drug challenge was available.

Both challenges and skin tests were conducted in the Allergy Unit, ensuring the presence of trained medical personnel and the availability of necessary medication and equipment to address potential anaphylactic reactions.

Patients received the treating physician’s contact details and were instructed to reach out if a suspected late reaction occurred at home. In addition, a phone survey was conducted five days after the oral challenge to make sure no late reaction was observed. 

### 2.3. Long-Term Follow Up

Long-term outcomes were assessed through review of electronic medical records (for antibiotic purchases post-evaluation with a specific focus on the suspected culprit drug) and structured phone interviews.

A thorough phone survey was administered to all participants in the study group. The objective of this survey was to augment the existing computerized data by ascertaining whether penicillin antibiotics were used subsequent to the evaluation. In cases where penicillin was indeed consumed, participants were asked if any adverse reactions were experienced. For those who refrained from using penicillin, their reasons for avoidance were explored. Additionally, participants were queried about their future intentions regarding the use of beta-lactam antibiotics if deemed necessary.

The utilization of penicillin was deemed affirmative if it was confirmed both through the phone survey and if evidence of purchase was found in the electronic medical file.

This study was approved by the local ethics committee.

### 2.4. Statistical Analysis

The results are expressed as frequency and percentage or mean and standard deviation, as appropriate. Differences between groups were analyzed using the chi-square test for categorical data, the *t*-test for continuous, normally distributed variables, and the Mann–Whitney U test for continuous parameters that did not have a normal distribution (for comparison between two groups). *p* values < 0.05 were considered statistically significant. Data were analyzed using SPSS-23 software (IBM SPSS, Armonk, NY, USA).

## 3. Results

Between 2009 and 2019, a total of 1860 elderly individuals sought care at the Allergy Unit of Meir Medical Center. Upon reviewing their medical records, we identified 270 patients (14.5%) who were investigated for suspected drug allergies. Of these, 166 patients (61.5%) were evaluated for suspected beta-lactam allergy. The remainder were referred for evaluation of allergies to various others substances, including NSAIDs (23, 8.5%), lidocaine (16, 5.9%), non-BL antibiotics (15, 5.5%), antihypertensives (3, 1.1%), carboplatin-based chemotherapy (15, 5.5%), biological therapies (5, 1.8%), steroids (6, 2.2%), proton pump inhibitors (PPI) (3, 1.1%), iodine contrast agents (6, 2.2%), and others. Demographic and clinical data are presented in Table 1. The mean age was 71 ± 9 (mean ± SD), with the majority being female (121, 72.9%). A significant portion (100, 60.2%) sought allergy investigation more than a decade after the alleged allergic reaction. Seventeen patients were evaluated for more than one beta-lactam agent. In total, 183 allergic reactions were documented (Table 1), with nearly half attributed to Penicillin VK (88, 48%). The most commonly reported major symptom was rash (105, 57.3%), followed by angioedema (28, 16.8%), dyspnea (16, 936%), abdominal pain (11, 6.6%), and hypotension (6,3.6%) (Table 2). Most patients (100,60.2%) sought consultation more than a decade after the alleged initial allergic reaction (Table 1).

The results of the allergic workup are presented in Table 2. Skin testing was conducted in 114 patients, yielding fourteen positive results (12.6%). Oral challenges were performed in 120 patients. Patients with a positive skin test (14), suspected severe delayed-type hypersensitivity, those suspected to have a severe delayed-type hypersensitivity (4), or histories suggestive of side effects rather than true allergic reactions (28) were excluded from the oral challenge. Three positive oral challenges (2.5%) were recorded, and two involved immediate reactions. The first patient had hives and angioedema and was treated with antihistamines and steroids. The second patient had an immediate cough and hives and was treated with inhalations of a beta-agonist and antihistamines. The third patient developed a late rash that resolved spontaneously. No severe reactions were noted, no patient required admission for further care or observation, and there was no need for emergent adrenaline treatment.

In total, a beta-lactam allergy was excluded in 145 patients (87.3%). Sixteen patients (9.6%) were diagnosed with an immediate type BL allergy, four patients (2.4%) with severe delayed-type hypersensitivity to BL, and one patient (0.6%) with a late benign rash.

Out of the 145 patients who were de-labeled, 106 were available for long-term follow up (3–13 years from evaluation, Table 3). Nineteen patients had passed away, and twenty patients were lost to follow up. Among those followed, thirty-eight patients (35.8%) were treated with the previously suspected culprit drug, and sixty-four patients (59%) expressed willingness to receive beta-lactam treatment if indicated necessary. Only four patients (3.7%) declined treatment with the culprit drug, despite clearance from the allergy evaluation. Among the thirty-eight patients re-exposed to beta-lactam antibiotics, three (7.8%) developed a late benign rash that resolved spontaneously after completing the antibiotic course. None reported immediate or severe delayed hypersensitivity reactions after exposure to the culprit drug.

## 4. Discussion

Falsely labeling patients with BL allergies has significant implications for morbidity and mortality, particularly in the elderly [17]. While the safety and efficacy of BL de-labeling in the general and pediatric population are well established [1,11,12,13], data on the elderly population are limited. This study demonstrates the efficacy and safety of BL de-labeling in a large cohort of elderly patients previously labeled with a BL allergy.

Elderly individuals are frequently labeled with BL allergies during their lifetime. In our study, approximately 15% of elderly patients who were referred to our clinic were evaluated for suspected drug allergies. Most of them, over 60%, were referred to our clinic for suspected “BL allergy” evaluation. This high rate of BL allergy labeling is likely partially attributed to the increased use of antibiotics and polypharmacy in this population [3]. Beta-lactam labeling has significant implications for both the general population and specifically for the elderly. These implications include the use of second-line antibiotics, resulting in more adverse drug events and a notably higher incidence of infections caused by multi-drug-resistant organisms [9,10]. Drug allergy labeling also adversely affects the quality of life in the geriatric population due to concerns about potential allergic reactions when prescribed medications [8]. Conversely, drug de-labeling, both in general and specific BL de-labeling, has been demonstrated to be effective and safe in the general and pediatric populations [1,11,12]. The de-labeling process involves a thorough clinical history, skin testing when indicated, and drug provocation tests, typically using the suspected drug [1,18]. In the elderly, obtaining a drug allergy history can be more challenging due to polypharmacy, memory decline, and the long latency of time from the original allergic reaction until the date of evaluation [3]. Furthermore, concerns have been raised regarding the risk of drug provocation in the elderly due to their fragility and presence of co-morbidities [2,3]. Consequently, it is not surprising that data show that physicians tend to avoid drug provocations in this population, even when they may be warranted [19]. *Wong* et al. found that an age over 75 was a significant factor in avoiding drug allergy testing in a large cohort of Asian patients [20]. *Pena-Acevedo* et al. also found that older age was a reason to avoid drug provocation in a cohort of 977 Spanish patients referred for drug allergy evaluation at an allergy clinic in Spain [21]. *Yildiz* et al. evaluated 175 elders with drug allergies, but the evaluation was conducted with anamnesis only, and skin tests or oral challenges were not administered, possibly due to concerns regarding the older age of the patients [5].

We describe here the results of a thorough allergy workup in 166 elderly patients with BL allergy labeling. The majority of patients in our cohort were female, possibly reflecting the longer life expectancy of females in Israel or due to the differences in healthcare-seeking behaviors between the sexes. Additionally, most patients were referred for allergy evaluation years after the alleged allergic reaction. This differs from our experience in evaluating NSAID allergies in the same population (60% were evaluated more than a decade after the reaction versus 30% in NSAID, *p* < 0.01, unpublished data), where patients tend to seek allergy evaluation within a year after the initial reaction. This is likely due to the availability of non-beta-lactam antibiotics and the reduced need for antibiotic use at younger ages when the allergic reaction occurred. This is opposed to the relative urgent need for NSAID allergy evaluation due to the lack of other available drug classes for pain and anti-inflammatory treatment. A lack of awareness among family physicians and the general population regarding the importance of BL allergy de-labeling likely contributes to the delayed referral and evaluation.

Penicillin VK was the most common culprit drug in our study, in contrast to general and pediatric populations, where amoxicillin typically predominates [11,16]. This discrepancy likely arises from historical factors, with Penicillin VK being most prevalent in the past, while amoxicillin is the more commonly used beta-lactam today.

As detailed in the Methods Section, our protocol for BL de-labeling included an extensive ST, which covered both major and minor determinants, as well as a direct oral challenge when indicated. Although using BL major and minor determinants for skin testing may result in false-positive results, we addressed this limitation by recommending an oral challenge in cases where the clinical history was not consistent with an immediate-type reaction, despite the positive skin test results. As mentioned earlier, since 2015, direct oral challenges were recommended when the clinical history suggested a delayed-type reaction.

As expected, a significant majority of patients did not exhibit a true beta-lactam allergy. Given that the OCT was performed with the culprit drug and that the majority of patients were not found to be allergic, it was not needed or possible to assess cross-reactivity between the different beta-lactam groups. De-labeling proved successful in over 87% of cases. This is in accordance with *Accarino JJ et al.*, who described successful BL de-labeling in 95% of 235 elders with BL allergy labels [14]. Notably, in 16% of patients, a detailed medical history alone was sufficient to exclude beta-lactam allergies. In the remaining cases, graded drug challenges were conducted after an immediate allergy was ruled out through anamnesis and skin tests, when deemed necessary.

Regardless of co-morbidity, fragility, or chronic medication use, drug provocation was administered to all eligible patients. A key finding of our study is that graded beta-lactam provocation, conducted after a thorough allergic evaluation that includes anamnesis and skin tests when immediate hypersensitivity was suspected, is safe, even in this vulnerable population. This safety profile was consistent among patients who underwent a direct oral challenge testing protocol (applied since 2015 in low-risk patients).

The incidence of allergic reactions during oral challenge testing was exceedingly low, and all reactions were mild, requiring no administration of adrenaline. These findings are in line with previous data from both adults and pediatric populations, reinforcing the safety of graded drug provocation testing following a comprehensive allergic workup [1,11,13]. In accordance with our results, *Epstein-Rigbi* et al. reported very low rates of systemic reactions in a large cohort of elders admitted to an internal medicine department who were prescribed BL antibiotics despite BL allergy labeling [22]. In contrast to their findings, we describe the safety of an oral graded challenge to the culprit BL antibiotic, as opposed to the safety of prescribing a general non-related BL. Importantly, our protocol proved not only safe but also effective. Long-term follow up revealed no cases of immediate reactions upon re-exposure to the culprit drug. Furthermore, the majority of patients expressed confidence in using beta-lactam antibiotics after undergoing the allergic evaluation. This stands in contrast to data in the general population, which indicates a high rate of patient reluctance to use beta-lactams, even after a favorable allergic workup [23].

This study has several limitations. First, it represents the experience of a single specialist center in Israel, and we do not have the incidence of BL allergy labeling or outcome in the general geriatric population. Nevertheless, our center functions as a tertiary center in Israel, and patients with drug allergies are referred to our center for evaluation from all across Israel, giving our findings a national perspective. Second, follow up was available for only a portion of our patients; most of them did not require the use of BL antibiotics during the follow-up period. Thus, data on patients lost to follow up or those who did not re-use beta-lactam remain unavailable. Despite this, the uniformly favorable outcomes observed among patients who were followed suggest that the approach is broadly safe and effective.

This study provides valuable evidence on the safety and efficacy of BL allergy de-labeling in the geriatric population, an area that remains underexplored. In the future, laboratory tests, such as the basophil activation test (BAT), may be validated for drug allergy evaluation, enabling us to predict drug allergic reactions and potentially eliminate the need for drug challenges, especially in vulnerable populations [24,25].

In conclusion, our findings highlight the widespread occurrence of falsely labeled beta-lactam allergies among the elderly. The de-labeling of a beta-lactam allergy proves to be both safe and effective in this demographic. Increased awareness is crucial for enhancing allergic workup in the geriatric population bearing beta-lactam allergy labels. We believe that establishing the removal rate of false BL allergy labels as a healthcare quality metric, and making it mandatory under the supervision of health authorities will improve outcomes for both the general population and the geriatric population.

## Figures and Tables

**Table 1:** Demographics.

Category	Data	N = 166
Age (mean ± SD)		71 ± 9
Gender (female)		121 (72.9%)
Time from allergic reaction to evaluation (years)	<1	52 (31.3%)
	1–10	11 (6.6%)
	>10	100 (60.2%)
Type of BL *	Penicillin VK	88 (48%)
	Amoxicillin	39 (21.3%)
	Amoxicillin–clavulanic acid	24 (13.1%
	Cefazolin	11 (6%)
	Cefuroxime	18 (9.8%)
	Ceftriaxone	3 (1.6%)

* Seventeen patients were allergic to more than one BL.

**Table 2:** Clinical evaluation.

Clinical and Allergy Evaluation		No (%)
Major clinical sign	Rash	105 (63.2%)
	Angioedema	28 (16.8%)
	GI signs	11 (6.6%)
	Hypotension	6 (3.6%)
	Dyspnea	16 (9.6%)
Initial clinical impression	Immediate reaction	40 (24.1%)
	Benign rash	96 (57.8%)
	Severe delayed reaction	4 (2.4%)
	Side effect	28 (16.8%)
Positive skin tests		14/114 (12.2%)
Positive oral challenge test		3/120 (2.5) *
Diagnosis	Immediate allergy	16 (9.6%) **
	Late benign allergy	1 (0.6%)
	Severe delayed allergy	4 (2.4%) ***
	No BL allergy	145 (87.3%)

* Two immediate reactions and one late benign rash were recorded in the oral challenge test; ** fourteen positive STs and two immediate reactions were found in the oral challenge test; *** a severe delayed allergy was based on anamnesis and STs, and oral challenges were excluded for these patients.

**Table 3:** Long-term follow up.

Long-Term Follow Up *		N = 106
	Used BL	38 (35.8%)
	Did not use BL but will use if indicated	64 (60.3%)
	Refuses BL use	4 (3.7%)
Outcome after BL re-use		N = 38
	Immediate allergy	0
	Late benign allergy	3 (7.9%) **
	Severe delayed allergy	0
	No BL allergy	35 (92.1%)

* Long-term follow up was performed 3–13 years after allergy evaluation; ** all cases were resolved spontaneously after the antibiotic course ended.

## Data Availability

Source data are available for review upon request.

## Abbreviations

BL	Beta-lactam
ST	Skin test
NSAIDs	Non-steroidal anti-inflammatory drugs
PPI	Proton pump inhibitor

## References

[1] Khan D.A., Banerji A., Blumenthal K.G., Phillips E.J., Solensky R., White A.A., Bernstein J.A., Chu D.K., Ellis A.K., Golden D.B. (2022). Drug allergy: A 2022 practice parameter update. J. Allergy Clin. Immunol..

[2] Cardona V., Guilarte M., Luengo O., Labrador-Horrillo M., Sala-Cunill A., Garriga T. (2011). Allergic diseases in the elderly. Clin. Transl. Allergy..

[3] Li P.H., Chung H.Y., Lau C.S. (2021). Epidemiology and outcomes of geriatric and non-geriatric patients with drug allergy labels in Hong Kong. Hong Kong Med. J..

[4] Ventura M.T., D’Amato A., Giannini M., Carretta A., Tummolo R.A., Buquicchio R. (2010). Incidence of allergic diseases in an elderly population. Immunopharmacol. Immunotoxicol..

[5] Yildiz E., Çölkesen F., Arslan Ş., Evcen R., Sadi A.F., Kilinç M., Aytekin G. (2021). Allergic diseases in the elderly population: A single-center experience. Turk. J. Med. Sci..

[6] Salkind A.R., Cuddy P.G., Foxworth J.W. (2001). The rational clinical examination. Is this patient allergic to penicillin? An. evidence-based analysis of the likelihood of penicillin allergy. JAMA.

[7] Shenoy E.S., Macy E., Rowe T., Blumenthal K.G. (2019). Evaluation and management of penicillin allergy: A review. JAMA.

[8] Blumenthal K.G., Harkness T., Phillips E.J., Ramsey A., Banerji A., Samarakoon U., Stone C., Fu X., Khan D.A., Otani I. (2020). Patient Characteristics and Concerns About Drug Allergy: A Report from the United States Drug Allergy Registry. J. Allergy Clin. Immunol. Pract..

[9] Macy E., Contreras R. (2014). Health care use and serious infection prevalence associated with penicillin “allergy” in hospitalized patients: A cohort study. J. Allergy Clin. Immunol..

[10] Blumenthal K.G., Lu N., Zhang Y., Li Y., Walensky R.P., Choi H.K. (2018). Risk of meticillin resistant Staphylococcus aureus and Clostridium difficile in patients with a documented penicillin allergy: Population based matched cohort study. BMJ.

[11] Confino-Cohen R., Rosman Y., Meir-Shafrir K., Stauber T., Lachover-Roth I., Hershko A., Goldberg A. (2017). Oral Challenge without Skin Testing Safely Excludes Clinically Significant Delayed-Onset Penicillin Hypersensitivity. J. Allergy Clin. Immunol. Pract..

[12] Iammatteo M., Lezmi G., Confino-Cohen R., Tucker M., Ben-Shoshan M., Caubet J.C. (2021). Direct Challenges for the Evaluation of Beta-Lactam Allergy: Evidence and Conditions for Not Performing Skin Testing. J. Allergy Clin. Immunol. Pract..

[13] Vezir E., Dibek Misirlioglu E., Civelek E., Capanoglu M., Guvenir H., Ginis T., Kocabas C.N. (2016). Direct oral provocation tests in non-immediate mild cutaneous reactions related to beta-lactam antibiotics. Pediatr. Allergy Immunol..

[14] Accarino J.J.O., Ramsey A., Samarakoon U., Phillips E., Gonzalez-Estrada A., Otani I.M., United States Drug Allergy Registry Study Team (2023). Drug Allergy in Older Adults: A Study from the United States Drug Allergy Registry. Ann. Allergy Asthma Immunol..

[15] Wurpts G., Aberer W., Dickel H., Brehler R., Jakob T., Kreft B., Mahler V., Merk H.F., Mülleneisen N., Ott H. (2020). Guideline on diagnostic procedures for suspected hypersensitivity to beta-lactam antibiotics: Guideline of the German Society for Allergology and Clinical Immunology (DGAKI) in collaboration with the German Society of Allergology (AeDA), German Society for Pediatric Allergology and Environmental Medicine (GPA), the German Contact Dermatitis Research Group (DKG), the Austrian Society for Allergology and Immunology (ÖGAI), and the Paul-Ehrlich Society for Chemotherapy (PEG). Allergol. Select.

[16] Confino-Cohen R., Rosman Y., Lachover I., Meir Shafrir K., Goldberg A. (2016). The Importance of Amoxicillin and Amoxicillin-Clavulanate Determinants in the Diagnosis of Immediate Allergic Reactions to β-Lactams. Int. Arch. Allergy Immunol..

[17] MacFadden D.R., LaDelfa A., Leen J., Gold W.L., Daneman N., Weber E., Al-Busaidi I., Petrescu D., Saltzman I., Devlin M. (2016). Impact of Reported Beta-Lactam Allergy on Inpatient Outcomes: A Multicenter Prospective Cohort Study. Clin. Infect. Dis..

[18] Rosman Y., Elmalak M., Meir-Shafrir K., Lachover-Roth I., Cohen-Engler A., Confino-Cohen R. (2021). Clinical history in suspected cases of immediate allergy to beta-lactam. World Allergy Organiz, J..

[19] Montanaro A. (2000). Allergic disease management in the elderly: A wakeup call for the allergy community. Ann. Allergy Asthma Immunol..

[20] Wong J.C.Y., Li P.H. (2021). Drug allergy management in the elderly. Curr. Opin. Allergy Clin. Immunol..

[21] Peña-Acevedo Y., Rosado-Ingelmo A., Vargas Porras W., Brandoni-Petrone M., Tejedor-Alonso M.A. (2023). Analysis of the safety of drug allergy workups in a spanish university hospital: Drug characteristics, type of reaction, and patients’ age at the initial assessment. Int. Arch. Allergy Immunol..

[22] Epstein-Rigbi N., Ziv S., Bulanenkova M., Bouganim R., Tal-Jasper R., Marchaim D. (2024). Beta-lactam antibiotics administration among adult inpatients with a beta-lactam allergy label: Incidence, predictors, and outcomes. Antimicrob. Steward. Healthc. Epidemiol..

[23] Lachover I., Sharon S., Rosman Y., Meir-Shafrir K., Confino-Cohen R. (2019). Long Term Follow up after Penicillin Allergy De-labeling in Ambulatory Patients. J. Allergy Clin. Immunol. Pract..

[24] Bogas G., Ariza A., Vázquez-Revuelta P., Labella M., Madrigal-Burgaleta R., Fernández-Santamaría R., Calvo-Serrano S., Villar-Chamorro E., Martín-Clavo S., Lebrón-Martín C. (2025). Basophil activation test is a complementary tool in the diagnosis of immediate reactions to platinum salts and taxanes. Allergy.

[25] Torres M.J., Doña I. (2024). Drug hypersensitivity: Past, present and future. Allergy.

